# Teacher Procrastination, Emotions, and Stress: A Qualitative Study

**DOI:** 10.3389/fpsyg.2019.02325

**Published:** 2019-10-11

**Authors:** Sara Laybourn, Anne C. Frenzel, Thomas Fenzl

**Affiliations:** ^1^Department of Psychology, University of Munich, Munich, Germany; ^2^Psychology School, Faculty of Economics and Media, Fresenius University of Applied Sciences, Munich, Germany

**Keywords:** procrastination, teacher procrastination, teacher emotion, teacher stress, qualitative analysis (QA)

## Abstract

Stress and negative emotions in teachers can lead to occupational burnout, poor performance in the classroom, and decreased job-satisfaction. Apart from having negative personal and physical effects for the individual teacher, teacher stress and burnout are also thought to have negative effects on the respective students and student achievement. As one potential source of teacher stress, procrastination has been speculated about. However, research on the phenomenology and prevalence of procrastination among teachers, as well as its relevance for their emotional and stress experiences, is very scarce. Further, most of the existing research on teacher procrastination used general self-report scales to obtain results. The present study therefore investigated the phenomenology of teacher procrastination as well as its links with emotional experiences and stress, using a qualitative approach. Twenty-seven male and female teachers from Germany were interviewed personally (Mean age = 35.7, SD = 9.64, Min = 25 years, Max = 67 years). Nine of those teachers reported to never needlessly delay an action concerning their profession or not to perceive their dilatory behavior as negative and stressful. Data from the remaining 16 teachers (Mean age = 35.06, SD = 7.01, Min = 26 years, Max = 48 years) were analyzed on the basis of qualitative content analysis by using deductive as well as inductive category application. Results revealed that these teachers procrastinate on an array of professional tasks, such as administrative and organizational tasks and correcting students’ work. The results showed that teachers delayed these tasks for different reasons but mainly due to task aversiveness. Further, teachers reported experiencing mainly negative emotions when procrastinating and perceiving their procrastination behavior as moderately stressful, indicating that procrastination is a potential stressor in the teacher profession. Limitations of the study are discussed and directions for future research are proposed.

## Introduction

Even though it is generally known that teachers need to abide by tight schedules and dense curricula and sometimes deal with highly challenging classroom and students’ home environment situations, there seems to be an implicit assumption in society that teachers have an easy job (Labaree, 2000; Moulthrop et al., 2006). This assumption may be due to the fact that teachers often have longer holidays than most other professions and – at least in Germany where half-day school is the norm – “free afternoons,” and because they are hardly ever monitored regarding their work. As such, the teaching profession is characterized by high degrees of self-determination; for large parts of their working days and years, they can work autonomously and at their own pace. Indeed, a large majority of teachers have been shown to choose teaching for intrinsic motives (e.g., Richardson and Watt, 2006, 2014) and thus teachers can be expected to be highly intrinsically motivated in their jobs. However, it may be that these working conditions in fact are problematic for some teachers, as for instance the high autonomy of the teaching profession also requires excellent self-regulation competencies (Kunter et al., 2013). Some teachers may have difficulties here, which, besides others, may result in a certain unwanted behavior often observed in student populations, namely procrastination. Contemporary definitions propose that procrastination entails a self-regulatory failure (Sirois and Pychyl, 2013; Anderson, 2016) in terms of voluntarily and needlessly delaying an intended action (Wohl et al., 2010) despite knowing or expecting to be worse off for the delay (Steel, 2007).

The overwhelming majority of past empirical research on procrastination has been accumulated based on student samples. There is only scattered initial empirical evidence suggesting that teachers are also at risk of procrastinating in their profession and that this leads to negative effects for the teacher, such as higher perceived stress (Verešová, 2013) and decreased job-satisfaction (Mohsin and Ayub, 2014). Decreased job-satisfaction in teachers and teacher stress have in turn been linked to occupational burnout (Montgomery and Rupp, 2005; Skaalvik and Skaalvik, 2009). Apart from having negative personal and physical effects for the individual teacher, teacher stress and burnout are also thought to have negative effects on the respective students and student achievement (Roeser et al., 2013). As such, scientific inquiry into teacher procrastination seems warranted.

### The Nature of Procrastination and Its Correlates

Procrastination is a complex phenomenon, making it somewhat difficult to distinguish from other similar concepts and often easy to mistake for something it is not, such as poor time management ability or laziness (Lay and Schouwenburg, 1993; Steel et al., 2001). The complexity of this phenomenon, which includes personal and situational factors as well as an interplay between the two, may also be the reason for the array of different approaches to and definitions of procrastination in the past (Klingsieck, 2013). For instance, some researchers have argued that delaying one’s intended actions has no further consequences or may even be beneficial for some individuals (e.g., Chu and Choi, 2005; Burka and Yuen, 2008; Abramowski, 2018). However, most researchers in the field today agree that there is no such thing as “functional” or “strategic” procrastination (see e.g., Anderson, 2016) because voluntarily, consciously, and needlessly delaying one’s intended actions while knowing one will be worse off due to the delay, implies a failure in self-regulation (Sirois and Pychyl, 2013; Anderson, 2016). This lack of self-regulation has been found to be predominantly maladaptive, as this behavior typically results in negative consequences for the individual. The consequences include increased perceived stress and poorer health (Tice and Baumeister, 1997; Sirois et al., 2003), depression (Lay and Schouwenburg, 1993), and experiencing negative emotions such as anxiety and shame (Solomon and Rothblum, 1984; Ferrari, 1991; Fee and Tangney, 2000; van Eerde, 2003).

While procrastination has been reported to be a dynamic behavior in certain situations (Moon and Illingworth, 2005) and to decrease slightly with age, it is generally thought to be stable over time and across contexts, indicating it to be a facet of a personality trait (Kim and Seo, 2015). But procrastination can also occur due to the task and situation at hand (Solomon and Rothblum, 1984). One reason for procrastination is thought to be the nature of the task. In an early study, Solomon and Rothblum (1984) conducted a factor analysis on the self-report and behavioral data of 342 university students to identify specific causes of procrastination. They found that task aversiveness, which means disliking the task at hand or finding it unpleasant, was an important factor leading to procrastination. Expanding those findings, Blunt and Pychyl (2000) differentiated various facets of task aversiveness (e.g., affect, personal meaning, autonomy), which can occur in different stages of a project. They found that each main component of task aversiveness and procrastination correlated positively in nearly all project stages. A meta-analytical review by Steel (2007) also revealed that task aversiveness played an important role in triggering procrastination. He found that the more individuals disliked a task because the task was perceived as unpleasant, boring, uninteresting, effortful, or anxiety arousing, the more they procrastinated. These results were also supported in a recent qualitative study by Grunschel et al. (2013) who asked 36 students in an open-ended format what they deemed as main reasons for their academic procrastination: most students (*n* = 30) named perceived task aversiveness.

It is important to note that task aversiveness does not stem solely from a task itself. It also depends on the person, whether they perceive a task as aversive or not and whether this then leads to procrastination behavior. In this study, we therefore refer to the term perceived task aversiveness to stress the complex interplay between a person and the environment in which the task at hand occurs (Moon and Illingworth, 2005).

In addition to task aversiveness, Solomon and Rothblum (1984) further showed that fear of failing at a task accounted for 49.4% of the variance in procrastination. In other words, people procrastinated on tasks that aroused their anxiety regarding not meeting their own or others’ expectations when working on them. Haghbin et al. (2012) also found fear of failure to be related to procrastination. More specifically, the authors found that this relation was moderated by self-perceived competence and mediated by need for autonomy.

Similarly, there is further empirical evidence suggesting that procrastination behavior is linked to poor competence beliefs or lack of self-efficacy. For instance, Ferrari et al. (1992) found a significant negative relationship between general self-efficacy and reasons for procrastination, as well as general self-efficacy and procrastination frequency among 319 college students. Meta-analyses also revealed moderate significant negative correlations between self-efficacy and procrastination (van Eerde, 2003; Steel, 2007). In the qualitative study by Grunschel et al. (2013) mentioned above, many (*n* = 20) of the students also reported poor competence beliefs, such as the perceived lack of study skills, to be a reason for their academic procrastination.

Furthermore, procrastination has also been explored from a motivational perspective, specifically through the lens of Self-Determination Theory (Ryan and Deci, 2000). For example, Senécal et al. (1995) analyzed the self-report data collected from 498 junior college students regarding their procrastination behavior, self-regulation abilities, and intrinsic and extrinsic motivation related to school activities. They found that procrastination was significantly negatively related to intrinsic motivation and correlated significantly positively with external regulation and amotivation. These findings are in line with Haghbin et al. (2012), who report that a lower sense of autonomy, leading to external regulation and amotivation, is negatively related to procrastination behavior. Similarly, Visser et al. (2018) concluded from their interview-study with 22 students that a lack of (intrinsic) motivation is especially problematic for high-level procrastinators, who found it difficult to regulate their dilatory behavior.

Finally, as mentioned earlier, procrastination can also be considered the consequence of (unsuccessful) self- and emotion regulation, as recently proposed by Sirois and Pychyl (2013) (also see Pychyl and Sirois, 2016). They argue that procrastination results from people’s impulse-driven attempts to up-regulate their immediate mood, which however in fact implies self-regulatory failure. For instance, having to perform an aversive task may lead to negative emotions, such as anxiety or frustration. For short-term hedonistic reasons, people strive to avoid the aversive task in order to feel better (Tice and Bratslavsky, 2000). Therefore, in order to repair their mood, they fail in persisting in doing the aversive task at hand and procrastinate. Sirois and Pychyl (2013) base their reasoning on studies conducted by Tice and Bratslavsky (2000) as well as Tice et al. (2001), where participants favored emotion regulation over behavioral self-regulation, which resulted in procrastination. Recent studies confirmed that self-regulation and emotion regulation indeed play a specific role in procrastination. In their qualitative study, Lindblom-Ylänne et al. (2015) found that procrastinators, as opposed to strategic delayers for instance, showed a specific profile where they lacked self-regulatory skills. Eckert et al. (2016) found that when students learned to regulate emotions in an adaptive way (e.g., tolerating and modifying negative affect), this resulted in reduced procrastination behavior. Related to this idea of procrastination resulting from short-term hedonistic strivings is Steel’s (2007) proposed concept of timing of rewards and punishments. This concept states that even though individuals might have serious intentions of doing a specific task at a certain time, they delay the intended task in preference of another activity. Evidence suggests procrastinators prefer short-term benefits, i.e., doing something immediately gratifying instead of the actual task at hand, to greater long-term gains and gratification (Steel, 2007, 2010).

### Existing Findings on Teacher Procrastination

As teachers do not have a regular nine-to-five profession with a fixed working place, need to work autonomously for the most part of their job, and receive little to no supervision, the probability of displaying dilatory behavior is high in the teaching profession. There is scattered empirical evidence suggesting procrastination is an issue for some teachers. Nguyen et al. (2013) found that educators belonged to a group of professions categorized by the authors as moderate procrastination jobs. Their subsample of 63 educators averagely scored *M* = 3.53 (SD = 0.73) on the Irrational Procrastination Scale (Steel, 2010; measures were scored on a 5-point scale). They concluded that teachers have a moderate risk of procrastinating in their profession, as compared to food servers (*N* = 22; *M* = 4.39; SD = 0.64) who were classified as “high,” and military officer leaders (*N* = 26; *M* = 3.16; SD = 0.85) who were classified as “low risk” professionals.

In regard to effects of procrastination on teachers, there are some initial findings suggesting that these are negative. Verešová (2013) explored 194 elementary school teachers’ procrastination (assessed by using the General Procrastination Scale by Lay, 1986) and their stress. She found that procrastination correlated significantly positively with stress and burnout. Specifically, when differentiating stress into cognitive, emotional, and social stress, procrastination correlated significantly with all three (cognitive stress and procrastination at *r* = 0.51; emotional stress and procrastination at *r* = 0.23; social stress and procrastination at *r* = 0.33). Findings obtained by Mohsin and Ayub (2014) support Verešová’s results. The authors examined the data obtained by self-report scales from 150 high school teachers and found procrastination to be significantly positively related to work-related stress. Further, they found a significant and strong negative relationship between procrastination and job-satisfaction (*r* = −0.63). Stress and lack of job-satisfaction in teachers, in turn, have been shown to be associated with health issues, such as burnout and poor teaching performance (Montgomery and Rupp, 2005; Skaalvik and Skaalvik, 2009; Klassen and Chiu, 2010; Hanif et al., 2011).

These few findings indicate that some teachers are at risk of procrastinating in their profession. But these results reveal little about what exactly may be triggering procrastination among teachers, and what effects it may have on them. The present study aims to take a first step toward filling in this knowledge gap.

### The Present Study

Generally, the teaching profession can be considered a psychologically and emotionally demanding profession, which is often associated with health risks due to stress and negative affect (e.g. Guglielmi and Tatrow, 1998). The peculiar situational context of the teaching profession seems to provide a potential arena for the phenomenon of teacher procrastination. The findings presented above suggest that if teachers display procrastination behavior, they may well become at risk of suffering negative consequences, such as increased perceived stress. This reasoning is well founded in previous research on procrastination; however, empirical evidence regarding causes and effects of procrastination predominantly stems from student samples. Due to the parallelism between the work environment of teachers and the study environment of students – in the sense of self-regulation affordances – it might be possible that teacher procrastination is similar to academic procrastination. However, even though students and teachers share the same academic setting, findings from student samples cannot be simply generalized to the teacher population. Teachers clearly have greater autonomy and opportunities for self-determination than students, while at the same time compared to students they are far less formally evaluated and thus get considerably less feedback. This implies less pressure in the sense of potential failure – yet also deprives teachers of the opportunities to obtain positive reinforcement if they invested effort.

While there is a rich literature body on student procrastination where both qualitative and quantitative methodologies have been employed, research on teacher procrastination is scarce and, so far, seems to exclusively rely on quantitative approaches, using general, context-unspecific self-report instruments. In the present study, we therefore chose a qualitative approach to explore teachers’ self-reported experiences regarding the phenomenon of procrastination, investigating the following research questions: Are teachers familiar with the phenomenon and do they report to engage in procrastinating behaviors? What are the reasons behind this dilatory behavior? How does it affect teachers emotionally? What consequences does it have for teachers? And finally, is procrastination experienced as stressful for teachers?

## Materials and Methods

### Sample and Participant Selection

As this study is exploratory, convenience sampling was used to recruit participants. Participation was voluntary and oral informed consent was obtained from all participants at the beginning of the interview. Interviews were conducted at the respective schools or at teachers’ homes.

Overall, 27 German primary and secondary school teachers (from *Grund-, Haupt-, Mittel-,* and *Realschule,* a well as *Gymnasium*) were interviewed. Sixteen participants were female. On average, participants were 35.7 years old (SD = 9.64, Min = 25 years, Max = 67 years) and had on average 8.7 years of teaching experiences (SD = 9.32, Min = 1 year, Max = 42 years).

The first question in the interview procedure (see in more detail below) involved confronting the participants with the term “procrastination” as well as the definition thereof as adopted in this study. Participants were asked to indicate if this phenomenon was familiar to them and if they ever engaged in such behavior. Eleven teachers reported to not ever engage in such dilatory behavior according to this definition.

The sample of the remaining 16 teachers who reported to display dilatory behavior consisted of 11 female and five male participants with age ranging from 26 to 48 years (*M* = 35.06, SD = 7.01). Years of teaching experience ranged from one to 20 years (*M* = 7.56, SD = 5.94). The large distribution of age and years of teaching is beneficial for the present study’s qualitative approach, as this may lead to a broad and diverse source of personal information and experiences. Problem-centered interviews were continued only with this subsample.

### Interviews and Procedure

The research reported herein was conducted in accordance with the ethical standards expressed in the Declaration of Helsinki and has received a formal waiver of ethical approval by the ethics committee of the Department of Psychology, LMU Munich.

In order to investigate teacher procrastination on a phenomenological level, this study used a qualitative approach by conducting individual interviews. Guideline-based interviews were developed according to the stepwise method outlined by Helfferich (2010). Hence, all teachers received the same questions whereby the actual wording and order of these questions varied slightly across interviews. The interview guide was piloted on two teachers, who were also recruited by convenience sampling, and revised before actual data collection commenced. The full interview guide can be found in the Supplemental Material to this paper. All interviews were carried out in German, personally, and by the same interviewer.

The interviews lasted between 8 and 23 min and were recorded by the voice recording application on a smart phone. All interviews were transcribed verbatim. Any names or specific locations mentioned during the interviews were replaced by the letters XY. Each transcript received a number to guarantee anonymity.

After the participants’ consent to conduct and to record the interview was given, they were encouraged to talk as freely and openly as possible. At the beginning of each interview, demographic information was obtained. Participants were then asked if they knew the meaning of the term procrastination and if they displayed this behavior. To ensure all participants were referring to the same meaning of procrastination, the term was defined by the interviewer at the beginning of the interview, who stated, “In this interview, we will be talking about procrastination behavior in teachers. Have you heard this term before? As there are many different definitions of procrastination, I will define the one we will be referring to during the interview: Procrastination is the voluntary, needless delay of an intended action despite knowing or expecting to be worse off for the delay, which occurs in a professional academic setting. Is this behavior familiar to you?” Next, participants were asked to name all the tasks within their profession they could think of on which they procrastinated and the subjective reasons for this behavior. Participants were then asked how they generally felt when they were procrastinating. Further, participants were required to recall a specific situation where they had procrastinated on a certain task and to recall what discrete emotions they experienced in the moment of actually procrastinating. They were then asked if they thought that their procrastination behavior had a negative, positive, or no consequence for them personally or for their career. Finally, a single quantitative item was included where participants had to rate whether they experienced their procrastination behavior as stressful on a rating scale ranging from 1 (*not stressful*) to 7 (*extremely stressful*).

### Analysis Strategy

Data analysis was carried out on the basis of qualitative content analysis proposed by Mayring (2014) using the open access web-application *QCAmap* (Mayring and Fenzl, 2014). The majority of the textual material was analyzed by using a deductive coding guideline. This technique, also referred to as deductive category assignment, requires the theory-driven construction of a coding guideline, consisting of category definitions, anchor examples, and coding rules (Mayring and Fenzl, 2014). Based on previous findings in the procrastination literature outlined in section “Introduction” of this article, deductive coding frames were developed for each main question of the interview. In addition to exact definitions and coding rules for all categories, anchor examples for each category were extracted from the pilot interviews as well as from the actual interviews and included in the coding frame (see Table 1 for an extract of the coding guideline). For each interview, every single text passage referring to one of the categories in the coding guidelines was assigned to the corresponding category.

**Table 1:** Extract of the coding guideline for reasons for teacher procrastination.

Category definition	Anchor examples
*D1: Perceived task aversiveness* Task is perceived as unpleasant, boring, effortful (Solomon and Rothblum, 1984; Steel, 2007)	It is time consuming; It is just cumbersome somehow; It is a horrible task, it is not any fun
*D2: Fear of failure* Fear/concern about not being good enough; poor performance; cannot meet own or other’s expectations (Solomon and Rothblum, 1984)	Maybe you put yourself under pressure because you want to do it really well
*D3: Extrinsic motivation* External pressure, hardly any autonomy, one does the task because it is expected from one, because one has to do it (Senécal et al., 1995)	Tasks donot make sense, a total scheme aimed at creating work

The responses to the interview questions “Do teachers know the term procrastination?” (yes vs. no), “How do teachers generally feel when procrastinating?” (positive vs. negative vs. neutral), and “What consequences do teachers think their procrastination behavior has for them personally and professionally?” (positive vs. negative vs. none) were categorized using a deductive approach where categories were mutually exclusive. The textual material referring to the following questions was categorized using a deductive coding frame where multiple categorization was allowed: “What reasons do teachers state for procrastinating on certain professional tasks?” [categories were (1) perceived task aversiveness (Solomon and Rothblum, 1984; Blunt and Pychyl, 2000; Steel, 2007), (2) fear of failure (Solomon and Rothblum, 1984; Haghbin et al., 2012), (3) extrinsic motivation (Senécal et al., 1995), (4) hedonistic reasons (Steel, 2007; Sirois and Pychyl, 2013), and (5) low competence beliefs (Ferrari et al., 1992; van Eerde, 2003; Steel, 2007)] and “What discrete emotions do teachers feel in the moment of procrastination behavior?” [categories were anxiety, guilt, depression, anger, joy, happiness, unhappiness, contentment, and shame; taken from emotion scales used for example by Pychyl et al. (2000) to describe negative or positive emotional states]. During the procedure of categorizing the interviews, additional categories were added inductively for example when a participant stated a new reason for their procrastination behavior not mentioned in the previously discussed literature. According to Mayring (2014), this mixed procedure is suitable in case the coding guideline, which has been developed based on theoretical considerations and findings from previous research, does not fully cover the contents of the entire textual material.

The data regarding the interview question “On which professional tasks do teachers procrastinate?” were analyzed using an inductive procedure (Mayring, 2014). As to the authors’ knowledge, there are no prior findings regarding the specific tasks on which teachers procrastinate, categories needed to be extracted from the textual material itself in a first step, using the content analytical technique of inductive category formation. In this procedure, we categorized all text passages in which “interviewees reported any possible task which they voluntarily and needlessly delayed” (selection criterion). In addition to such a selection criterion, the specification of a level of abstraction, on which categories are phrased, is required for the inductive procedure (Haberfellner and Fenzl, 2017). In this study, categories were formulated as “specific tasks where procrastination behavior occurred” (level of abstraction). In a second step, a deductive coding guideline including anchor examples and coding rules was developed based on the inductive categories found in the first step, thus achieving a more transparent coding approach.

For all text analytical steps, the coding unit, which is the smallest component of the material that can be coded (sensibility), was set to a clear meaning component in the text. The context unit, which serves as the background for the coding decision, was the respective interview. By definition, the recording unit is set to all documents for inductive category formation and to the single document in deductive category assignment (Mayring, 2014). According to the step-by-step models for the various techniques of qualitative content analysis, all coding guidelines were revised if necessary during categorization of the textual material (Mayring, 2014; see Supplementary Material for full coding guideline).

Quantitative data were analyzed applying descriptive statistical procedures using *IBM SPSS Statistics 24* for *Windows*. Coding reliability was determined and frequencies were calculated for all answers and categories.

### Coding Reliability

In order to establish the degree of reliability for the category systems and reproducibility of the categorizations, a second researcher (intercoder) categorized a subsample of two randomly chosen interviews independently from the primary coder. The intercoder received the uncategorized text material, the coding guideline, and the content analytical rules. In the conclusive coding conference, the coded text material of the primary coder and the intercoder was used to compare each marked text passage and its assigned category for consensus.

Two different strategies of analysis were applied to obtain reliability. Two questions of the interview guide met the assumptions for Cohen’s Kappa (e.g., responses are measured on a nominal scale and categories are mutually exclusive). The intercoder agreement in terms of the percentage of agreed and non-agreed text passages was obtained separately for all remaining interview questions (Haberfellner and Fenzl, 2017). All items reached full or high agreement (*K* = 1.0; 91.7–100.0%) between the raters. Overall, we concluded that our category systems were highly reliable and our categorizations of the textual material highly reproducible.

## Results

### Teacher Procrastination in the Present Sample

Out of the 27 teachers initially recruited for the study, 11 reported not to procrastinate according to the study’s definition based on Steel (2007), Wohl et al. (2010), Sirois and Pychyl (2013), and Anderson (2016).

Among those 11 teachers, four simply stated never to delay work-related tasks. Two teachers reported to completely avoid delaying work-related tasks, as they perceived this as extremely unpleasant.

Four teachers reported to display some dilatory behavior but not to perceive it as stressful or not to expect to be worse off for the delay. Finally, one teacher explained that even though they might be initially angry with themselves for not starting work-related tasks earlier, they did not perceive their dilatory behavior as stressful as they needed some degree of pressure in order to work faster and efficiently. Specifically, that teacher stated:

I need that (*the pressure*). I’ve noticed that I can work very well shortly before a deadline; I do all the corrections then. I even have the feeling that I work more consistently and faster and that I can shut out everything else (Teacher 3, para. 58).

The remaining 16 teachers reported to procrastinate according to the present study’s definition of teacher procrastination. Interviews with them were continued to further explore which tasks they reported to procrastinate, what the reasons were, and which consequences procrastination had for them, particularly with respect to their emotional and stress experiences.

### Professional Tasks Regarding Teacher Procrastination

Professional tasks on which 16 teachers reported to procrastinate were summarized into four main categories: correcting students’ work, administration and organization, preparing lessons, and evaluating students on their general work and performance.

Delaying working on administrative and organizational tasks was mentioned most frequently (11 teachers). This included organizing field trips, structuring one’s paper work and lessons, and organizing parent-teacher conferences.

Correcting and evaluating students’ work was mentioned by 10 teachers in our sample. For the majority of teachers, this entailed evaluating and correcting students’ written exams. For elementary school teachers, correcting students’ work also involved evaluating students’ homework.

A further task mentioned by seven participants was evaluating students on their general work and test performance. Here, secondary school teachers reported procrastinating on grading tests as well as writing up report cards at mid-term and at the end of the year. Elementary school teachers also mentioned procrastinating the required daily or weekly evaluations of students’ behaviors and writing up narrative evaluations, which replace letter grades at the early elementary years.

Further, six teachers also reported to procrastinate on preparing and structuring their lessons. Others, however, reported to never procrastinate on tasks directly related to or involving the students, such as lessons. For example, one teacher said:

Everything I do personally for the children and for lessons I just do it because I know that I have to do it and I know that the children need it (Teacher 4, para. 19).

### Reasons for Teacher Procrastination

Teachers’ answers regarding the reasons for their procrastination were grouped into six main categories. The main reason for procrastinating on these tasks was perceived task aversiveness (stated by 13 teachers), that is finding the task uninteresting, boring, or effortful. This is illustrated by the following two examples:

Because I just don’t like doing it. Because it is a lot of work and yes…. Because when you have about 29 exercise books lying there in front of you and well yes… it is just very cumbersome (Teacher 13, para. 66).

Another reason for teacher procrastination was related to extrinsic motivation (stated by six teachers). The teachers reported procrastinating on tasks that lacked personal meaning but were often expected from them either by their principal or the ministry of education. For example, one teacher described this aspect in the following way:

Well, you really have to do so many things because it is just expected from you. And of course this somehow adds to the fact that you delay things, because you then think I am not doing this because I think that it makes sense, I am doing this because someone up there thinks we teachers have to do this on top of everything else (Teacher 1, para. 46).

Working conditions at the respective schools were also reported as one reason for procrastinating on school-related tasks (stated by six teachers). This category emerged inductively from the interview data and was added to the respective coding guideline during revision. It appears that teachers do not always have fixed working places at their schools where they can store and leave material needed for lessons, such as books, or where they can work quietly for a longer period of time. Further, teachers reported not having access to a computer at work where they can do research or design exercise sheets. Moreover, elementary school teachers expressed that they were missing some kind of technical support in writing and organizing daily or weekly student evaluations. The respective teachers stated that these circumstances led them to doing the required work for their profession at home, where they are more likely to engage in procrastination behavior. As an example, one teacher reported the following:

On the other hand I always say to myself that I am only being provided with the means that are there at the moment. So, if someone wants me to prepare my lessons in a way that they will work, so that then… well maybe not necessarily on the level of a lesson demonstration but that they are prepared in such a way which it is supposed to be nowadays,… then the infrastructure, that’s what I’m going to call it, would need to be different (Teacher 16, para. 33).

Four of the 16 interviewees reported to procrastinate also due to what we label “hedonistic reasons” here (referring to reasoning proposed by Sirois and Pychyl, 2013; and Steel, 2007). Teachers reported intending to start and finalize work-related tasks a lot sooner but then deviated from their initial intentions and giving in to more immediate pleasant tasks, as the due date was still further in the future. However, as the due date came closer, teachers reported to feel uncomfortable for initially delaying the task. Two teachers also reported often doing pleasant activities first before preparing the lessons for the next day, even though they intended otherwise and their dilatory behavior meant working late.

Three teachers indicated that their procrastination behavior was due to their poor competence beliefs. One teacher reported frequently delaying writing letters to parents, as they did not feel competent enough to write them adequately. Further, teachers reported to procrastinate on preparing for a certain lesson, as they did not feel competent enough to teach that subject. For example:

I think there is this feeling involved… such as yes, I cannot do that anyway. So like… what is that? Not believing in oneself or something like that? (-) Yeah, because you simply don’t trust yourself to do the task at hand (Teacher 1, para. 43).

Two teachers reported procrastinating on professional tasks also due to fear of failure. Here, fear of failure touched on the concern not to meet one’s own or others standards: “But maybe you put yourself under pressure then because you want to do it especially well…” (Teacher 11, para. 40).

### Emotional Experience Regarding Teacher Procrastination

Fifteen teachers reported feeling overall negatively when procrastinating. When asked to state discrete emotions they felt at the moment of procrastination, teachers reported a variety of negative emotions such as feeling angry (stated by nine teachers), guilty (four teachers), or unhappy (two teachers) but also disappointed (two teachers). The latter emotion emerged during the interview process and was inductively added to the respective coding guideline during revision. Five participants were not able to name a specific emotion but described feeling overall negatively. Therefore, the category undefined negative affect was added to the coding scheme. The following example illustrates this category:

But when I think about it or when the pressure starts to get stronger then it just blocks the happiness, the high spirits or the spontaneity or so. All of that is restricted. Then I think I should really be doing this. I have a feeling as if the spiral is turning further and further downwards and always… the noose tightens more and more (Teacher 11, para. 49).

One teacher reported feeling mixed emotions when procrastinating: joy for delaying and therefore not having to do the task at that moment, but at the same time a little dissatisfaction, as the task was still pending.

### Consequences of Teacher Procrastination

Regarding the question, whether procrastination had any consequences for the teachers personally or professionally, one teacher could not provide an answer. Seven of the remaining 15 teachers reported their procrastination behavior had negative consequences for themselves. The consequences were reported to be losing confidence in one’s abilities, having to forego doing other more pleasant things due to the previous procrastination behavior, and experiencing negative emotions, as illustrated in the following example:

One possible consequence is that on some days or even weeks I have to neglect everything else… Like not having time for my spouse or having to postpone personal activities. That is a pity then (Teacher 14, para. 50).

Two teachers reported that their procrastination behavior had positive consequences for them personally. One teacher saw suffering from dilatory behavior as a chance to better oneself and stop delaying work. The other teacher said they had learned from their procrastination that even though work was frequently delayed, in the end they always managed to complete it. This teacher reported to regard the behavior as a part of the teacher profession, which needed to be accepted as such.

The remaining six teachers reported not having any consequence from their procrastination behavior, neither for them personally or for their careers.

### Stressfulness of Teacher Procrastination

Overall, teachers in this sample perceived their procrastination behavior as moderately stressful [*N* = 15; *M* = 4.63; SD = 1.06; Range: 2–6 on a rating scale ranging from 1 (*not stressful*) to 7 (*extremely stressful*)]. Six teachers reported that their perception of their procrastination behavior (and with that the degree of perceived stressfulness) often changed depending on what stage of working on a task they were in. Two teachers reported that even though they felt negatively and stressed when procrastinating, overall they perceived the stressfulness of their dilatory behavior as low (level 2 of rating scale). One teacher could not provide any single score on overall stressfulness. This teacher stated:

That depends on the phase. Of course it’s not stressful at all at the time when I say okay, I am not doing that now. Then it’s great, of course, and it lets me have a lot of freedom in the teaching profession. Knowing that sometime along the way I will experience a level 7 of stress (Teacher 19, para. 26).

## Discussion

### Teacher Procrastination in the Present Sample

The aim of the present study was to gain deeper qualitative insight into procrastination among teachers. Overall, 27 teachers were interviewed. Of those, 11 participants reported to never procrastinate according to the study’s definition based on Steel (2007), Wohl et al. (2010), Sirois and Pychyl (2013), and Anderson (2016). Due to the focus of the study, these teachers’ responses were not analyzed further and any statements regarding why these teachers do not engage in dysfunctional dilatory behavior or in what way they differ from our definition of teacher procrastination are purely speculative. However, during the interviews it was apparent that these teachers did not (or thought they did not) engage in procrastination for different reasons, such as not wanting to feel stressed or to avoid experiencing negative emotions. Some of these teachers seemed to display dilatory behavior in their profession but reported not to be stressed or negatively emotionally aroused by this. One teacher claimed they needed the pressure to complete their tasks. It is not clear whether this teacher really does work better under pressure or whether they are deceiving themselves in thinking this is the case. Objectively, they actually could be procrastinating (Anderson, 2016). Future research may want to investigate possible differences between teacher procrastination and other dilatory behavior as well as the respective underlying reasons and objective consequences.

However, 16 out of the 27 teachers in our sample did report to regularly engage in procrastination behavior, which overall made them feel negatively and moderately stressed. In the following, we discuss our findings from the continuing interviews with those teachers.

### When and Why Teachers Procrastinate

The teachers in this study reported a variety of professional tasks on which they procrastinated. The most frequently reported tasks were working on administrative and organizational tasks, correcting students’ exams, and evaluating students’ overall performance.

With respect to our second research question, the reasons given by the teachers for procrastinating on these tasks corresponded with those discussed in the procrastination literature, namely (in decreasing order based on our sample findings) perceived task aversiveness, extrinsic motivation, adverse situational conditions, hedonistic reasons, poor competence beliefs, and fear of failure.

Regarding task aversiveness as a driver of procrastination, prior research (Solomon and Rothblum, 1984; Blunt and Pychyl, 2000; Steel, 2007) has reported significant yet moderately sized relationships between task aversiveness and procrastination behavior. When asked directly in interviews however, most students reported that task characteristics played a major role in their academic procrastination, especially when the task was perceived as aversive, complex, or stressful (Grunschel et al., 2013). The findings by Grunschel et al. (2013) are in line with our results. Perceived task aversiveness was clearly the most frequently reported reason for procrastination by the teachers in our study. The discrepancy between quantitative and qualitative results regarding the role of task aversiveness in procrastination may be due to the complexity of the task aversiveness construct. In quantitative research, task aversiveness can be measured specifically in all its dimensions (e.g., Blunt and Pychyl, 2000) by using adequate scales. By doing so, task aversiveness can be teased apart from other constructs. In qualitative studies, participants may use the term task aversiveness more broadly and, in some cases, potentially inappropriately. For example, if teachers are fearful of holding a lesson in a subject they are not comfortable with because they may seem incompetent in front of the class (i.e., fear of failure, poor competence beliefs), they may attribute the irrational delay to disliking the task instead of recognizing that they are afraid of failing. As such, our participating teachers may have been overreporting task aversiveness due to self-deceptive reasons. They may have not wanted to admit to the interviewer or themselves that they actually doubted their competencies and so reported to procrastinate because of disliking the task. Indeed, in our study, only two teachers explicitly mentioned fear of failure as reasons for their own procrastination behavior, and only four mentioned aspects of lack of competence as reasons. Further, we propose that task aversiveness is necessary, yet not sufficient for procrastination to occur. In other words, aversive tasks are not always procrastinated, but if tasks are procrastinated, they tend to be considered aversive by the actor. Future research may want to examine the nature and role of task aversiveness in teacher procrastination in more detail.

Nevertheless, the comparably low frequencies of fear of failure and lack of competence beliefs in our study also seem reasonable given that teachers are evaluated only very rarely. Much of the existing literature on procrastination focused on students who almost constantly write tests and are being graded, with potentially severe consequences on their future education and also careers (Kuncel et al., 2004). Therefore, students are under a great amount of pressure to do well during their education, which seems to render fear of failing at tasks, such as exams, an important reason to procrastinate. In contrast, in Germany, where the current study was conducted, teachers are evaluated much less frequently, their competence is rarely formally questioned, and harsh consequences of poor performance barely exist. Therefore, it is highly reasonable that competence doubts and fearing failure on professional tasks are less of an issue for them.

A further reason for procrastination reported by the teachers in this study was related to extrinsic motivation due to feeling externally regulated (Ryan and Deci, 2000). At the beginning of the interviews, many teachers mentioned that one major advantage of their profession was that they could choose when to work on certain tasks and to be free in making many decisions, for instance how to structure their lessons. However, as the interviews persisted, it became clear (also to the teachers themselves) that teachers felt somewhat restricted in what they could and could not do, as they have to follow strict curricula and perform a range of administrative tasks. As such, some teachers felt they had to complete certain tasks imposed on them by the ministry of education or their principals. Teachers reported to procrastinate on these tasks, specifically as they were perceived as being meaningless. These findings are in line with previous evidence indicating that procrastination is related to less autonomous forms of motivation (Senécal et al., 1995) and the need for autonomy (Haghbin et al., 2012).

Furthermore, one specific reason for teacher procrastination, which we had not anticipated from the existing literature and therefore emerged from the interview material, was adverse situational conditions. Six teachers in our sample reported to procrastinate due to adverse working conditions at their respective schools. On the one hand, they felt that if they had appropriate and individual working places equipped with storage places and computers or other technical support to organize for example student evaluations, they would procrastinate less. Due to the situational circumstances however, teachers reported to accomplish most of their school-related work at home, where they perceived the chances of procrastinating as higher. On the other hand, some teachers also stated that they procrastinated due to having too many duties in their profession. According to Dorsemagen et al. (2013), actual school lessons only account for 40% of teachers’ working hours. Therefore, more than half of teachers’ working hours are spent on other tasks such as preparation, corrections, evaluations, and administrative or organizational tasks. These tasks were mostly named by the participants of this study when asked on what professional tasks they procrastinated. Moreover, the circumstances in which teachers need to work and perform as well as their workload are likely to have an influence on teacher motivation, which in turn may again lead to procrastination behavior. These adverse working conditions thus seem to play a major role in teacher procrastination. At this point, our contextualized, qualitative, and open-ended methodology thus revealed important new insights.

Finally, four teachers reported to regularly give in to doing more pleasant tasks or activities and avoid doing unpleasant work-related tasks, even though they initially intended to do differently. These findings are in line with the idea that procrastination is a form of self-regulatory failure as proposed by Sirois and Pychyl (2013) in their mood repair model of procrastination, as well as the concept of timing of rewards and punishments as proposed by Steel (2007). In this respect, the teachers in our study reported very similar behaviors as has been shown for students in earlier research, namely putting their present self’s needs (i.e., avoiding the aversive task in order to feel better) above the future self’s needs (Sirois and Pychyl, 2013), especially when the anticipated consequences (i.e., punishments, such as feeling stressed) of not doing the work-related task were far into the future (Steel, 2007).

### Emotions and Perceived Stressfulness

To answer the research questions on how procrastination behavior affects teachers emotionally and to what extent dilatory behavior is perceived as stressful, the present study investigated teachers’ reported emotions experienced at the moment of procrastination through a qualitative approach and gathered a quantitative rating on how stressful teachers experienced their dilatory behavior. Past research has shown that habitual procrastination correlates positively with negative emotions. When explored through experience, sampling dilatory behavior was not found to correlate with either negative or positive emotions at the actual moment of procrastination (Pychyl et al., 2000). Therefore, we had explicitly asked our participants to report about the emotions experienced *at the moment of procrastination*. In total, 15 out of 16 teachers stated that their dilatory behavior made them feel negatively. Specifically, teachers reported experiencing a range of unpleasant emotions, such as guilt or unhappiness, in the moment of procrastination. Only one of our teachers reported a positive feeling at the moment of procrastination (though mixed with dissatisfaction). So overall, at least in retrospect, the emotional experiences that go along with procrastination are clearly predominantly negative.

Another interesting finding was that more than half of the participants in our study reported feeling angry when procrastinating. The feeling of anger was either directed at themselves for procrastinating in the first place, or because of the situation or task at hand. There is evidence that experiencing anger influences the vulnerability to illness (Suinn, 2001) and is related to higher stress and lower psychological well-being (Maan Diong et al., 2005). To the authors’ knowledge, anger has not been concentrated on distinctly in procrastination research. As stress and poorer health in teachers have been linked to procrastination, which in turn leads to absence and drop-out, future research should turn its attention to the role of anger in procrastination in general, but also specifically among teachers.

Finally, as judged from our single quantitative item incorporated toward the end of our interview guide, teachers on average reported feeling moderately and not highly stressed by their behavior, with mean ratings of below 5 on the 7-point scale ranging from *not stressful* to *extremely stressful*. At first glance, this may seem somewhat surprising, as the majority reported experiencing strong negative emotions when procrastinating. Then again, as participants also reported to not have suffered any major consequences due to their dilatory behavior (see in more detail below), on the whole, procrastination seems to be only moderately stressful for teachers. Yet overall, the results obtained in this study support previous findings that procrastination should be viewed as a potential source of stress in teachers’ lives (Verešová, 2013; Mohsin and Ayub, 2014). As stated before, teachers’ stress can lead to an array of negative personal, physical, and psychological consequences for the individual teacher, such as poor health and poor teaching performance (e.g., Skaalvik and Skaalvik, 2009). This in turn can influence students’ education and academic performance and their future careers (Roeser et al., 2013). We conclude that teacher procrastination as a potential stressor in teachers’ lives deserves further research attention.

### Consequences of Procrastination

With respect to our research question regarding consequences for the individual, all teachers agreed that their procrastination behavior had no severe consequences for their careers or for them personally as they either managed to finish their tasks on time or were able to hide the fact that they had procrastinated, through improvisation for example. Seven teachers reported that the consequences of their dilatory behavior had negative effects for themselves, such as experiencing negative affect or stress. Two teachers reported having positive consequences from procrastination, as they either saw it as a chance to better themselves or to accept it as part of the teaching profession. This is an interesting finding, as both teachers reported their procrastination behavior as being moderately stressful, and leading to experiencing negative emotions, such as depression and unhappiness. Viewing the consequences as positive and therefore distracting oneself from the possible issue at hand may be a coping strategy regarding their procrastination tendencies. Chu and Choi (2005) found evidence that procrastinators engaged in more avoidance-coping strategies than non-procrastinators. Therefore, procrastinators are more likely to ignore or distract themselves from the consequences of their procrastination behavior.

Seven teachers reported not having any consequences from their procrastination behavior, as they reported always finishing everything on time. This finding reflects the circumstances that teachers are not evaluated or directly monitored. Even if their procrastination leads to poorer performance, nobody apart from maybe the students will notice it. This may be another explanation why the participants in this study perceived their procrastination as only moderately stressful: Even though they procrastinate and it affects them negatively, there are no further objectively adverse consequences.

### Limitations and Implications for Future Research

The present study provided qualitative evidence of the construct of teacher procrastination on a phenomenological level. Nevertheless, the current findings should be interpreted with caution due to some limitations.

Findings in this study were obtained by conducting qualitative interviews and allocating teachers’ responses to categories derived by the authors from the procrastination literature as well as from the interview material itself. Even though the authors chose a rule-guided and systematic approach to textual analysis using Qualitative Content Analysis (Mayring, 2014) and took caution to be objective and transparent, categorization of textual material remains a subjective procedure due to the interpretative paradigm of qualitative research. Further, the findings in this study were exclusively obtained through self-report. Although this method allows detailed descriptions of teachers’ procrastination behavior and respective emotions, a disadvantage of this method is that the researcher is dependent on the participants’ ability and willingness to explain their experiences and feelings. Further, some of our questions required the participants to retrospectively report their experiences and feelings, which can be affected by memory biases. Moreover, the results obtained by the personal interviews may have been subject to self-deception and social desirability and therefore distorted, as the teachers may not have wanted to seem unprofessional by reporting a lot of procrastination behavior. Future research on teacher procrastination may use alternative methodological approaches such as state-based measures (experience sampling, see e.g., Pychyl et al., 2000), or behavioral trace data, to avoid this issue. In addition, we had included one single quantitative item in our study in order to explore the degree to which the teachers experienced their procrastination behavior as stressful. Single items clearly suffer from limited validity and reliability, yet research has shown that they can be highly effective for the assessment of affective content (Gogol et al., 2014), and the one-on-one interview context likely improved the conscientiousness with which teachers responded to this single item (as compared to a long questionnaire that is filled in individually). Nevertheless, future research should replicate and extend our findings based on this single item that teacher procrastination implies moderate levels of stress for teachers.

Due to the qualitative nature of the study, the sample was small and therefore the obtained results cannot be generalized across a larger teacher population. However, our qualitative approach allowed for an initial insight into the relatively unknown construct of teacher procrastination as defined in this study and provides the basis for further qualitative and quantitative research on teacher procrastination. For instance, working conditions were often reported as being one main reason for procrastination by the sample. Future research is needed to investigate how large the impact of working conditions is on teacher procrastination. As teacher procrastination has been linked to stress, optimizing teachers’ working places may be an affordable and effective way of reducing teacher procrastination and thus their stress experiences. As such, this finding may be interesting not only for future research but also schools and governmental bodies regarding how to structure the teaching profession in order to eliminate potential stressors.

A further implication for future research would be beneficial for understanding teacher procrastination as well as any other type of procrastination: past research has mainly comprised positive and negative emotion scores when investigating affect in regard to procrastination. The present study, however, found evidence that individuals experience an array of negative emotions when procrastinating. Especially, anger was mentioned most frequently. Exploring in more detail which discrete emotions individuals go through in regard to their procrastination behavior may prove fruitful for gaining deeper insight into this complex construct.

Previous research has found that procrastination is relatively stable over time and correlates inversely with conscientiousness (e.g., Steel, 2007; Kim and Seo, 2015), indicating it to be a facet of personality. However, situational-context factors can also lead to this behavior (Solomon and Rothblum, 1984). Our study design largely focused on the trait aspect of procrastination as we asked participants to report about their “general experiences” with respect to procrastinating. As such, our study does not provide insights into teacher procrastination as viewed from a state perspective. Future research could focus on this aspect as well.

Lastly, as students are regarded as a population high at risk for engaging in procrastination (Milgram et al., 1992), the question arises if teacher procrastination may influence student procrastination regarding their academic tasks. Procrastination research traditionally concentrates on researching the differences between individuals regarding their procrastination behavior. To the authors’ knowledge, there is no research on whether and how individuals affect one another regarding their procrastination behavior. There is substantial evidence that teachers have a great effect on their students, as they spend a lot of time together and are part of students’ social and cognitive development (Davis, 2003). Therefore, teachers’ procrastination behavior may influence their students’ procrastination behavior in either a positive or a negative way. Future research should investigate this possibility.

### Summary and Conclusion

With this study, we had set out to explore the phenomenon of procrastination especially for the population of teachers, which so far seemed largely underexplored. Given that the scarce existing literature that did address teacher procrastination in the past exclusively used quantitative approaches and general closed-ended, non-context-specific self-report instruments, we deemed a qualitative approach as valid and promising here, to take an open-ended perspective and regard the phenomenon directly from the teachers’ point of view. Our results showed that while the existing overarching conceptual frameworks proved basically applicable for teachers, past empirical findings – derived predominantly from student populations – proved not to be fully equivalent with our findings. Specifically, our study revealed that in comparison to students, lack of competence and fear of failure seem to play a less important role for procrastination among teachers. Instead, lack of meaning and corresponding extrinsic motivation for certain tasks required from the teachers seem to play a more important role than anticipated, as the teaching profession is known to be characterized by a high degree of self-determination and the majority of teachers have been reported to be basically highly intrinsically motivated for their job. Finally, while procrastination did bring about a range of negative emotions for teachers, specifically anger, and also guilt and disappointment, overall they reported that their procrastination behavior was only moderately stressful for them, which likely was due to the fact that they also experienced no major negative consequences of their dilatory behavior.

## Data Availability Statement

The datasets generated for this study are available on request to the corresponding author.

## Ethics Statement

The studies involving human participants were reviewed and approved by Ethics committee of the Department of Psychology, Ludwig-Maximilians-University Munich. Written informed consent for participation was not required for this study in accordance with the national legislation and the institutional requirements.

## Author Contributions

SL and AF contributed to conception of the study. SL, AF, and TF contributed to designing the methodology of the study. TF contributed to designing the relevant software. Data curation, formal analysis, and investigation were performed by SL. Project administration and writing the original draft were carried out by SL. All authors contributed to manuscript revision, read and approved the submitted version.

### Conflict of Interest

The authors declare that the research was conducted in the absence of any commercial or financial relationships that could be construed as a potential conflict of interest.
