# Supplementary material for: Teacher Procrastination, Emotions, and Stress: A Qualitative Study
Source: Front Psychol. 2019 Oct 11;10:2325. doi: 10.3389/fpsyg.2019.02325 (PMC6798067; doi:10.3389/fpsyg.2019.02325)
Supplement: Supplementary file 1 [file Data_Sheet_1.docx]

Supplementary Material

**Full Coding Guideline**

**Do teachers know the term procrastination?**

| **Category Definition** | **Anchor Examples** | **Coding Rules** |
| --- | --- | --- |
| B1: Approval  Positive answer regarding the question; the participant knows the term | Yes | Clear assignment; Responses mutually exclusive |
| B2: Rejection  Negative answer regarding the question; the participant does not know the term | No | Clear assignment; Responses mutually exclusive |

**On which professional tasks do teachers procrastinate?**

| **Category Definition** | **Anchor Examples** | **Coding Rules** |
| --- | --- | --- |
| C1: Correcting Students’ Work  (such as homework or written exams) | Correcting exams | Clear assignment; Multiple responses allowed |
| C2: Administration & organization | Checking first aid boxes; filing exercise sheets; Organizing outings;  Making calls; Entering grades in to the system | Clear assignment; Multiple responses allowed |
| C3: Preparing Lessons  Preliminary work for lessons and courses | Making „Reading Houses“ for the students | Clear assignment; Multiple responses allowed |
| C4: Evaluating Students on their General Work and Performance | Observing students and putting that to paper, which can be quite a big task;  Writing report cards | Clear assignment; Multiple responses allowed |
| C5: Developing Exams | Sometimes developing exams | Clear assignment; Multiple responses allowed |

**What reasons do teachers state for procrastinating on certain professional tasks?**

| **Category Definition** | **Anchor Examples** | **Coding Rules** |
| --- | --- | --- |
| D1: Perceived Task Aversiveness  Task is perceived as unpleasant, boring, effortful | Because it’s time consuming;  And it is just cumbersome somehow;  Because it’s a horrible task, it isn’t any fun | Clear assignment; Multiple responses allowed |
| D2: Fear of Failure  Fear/ Concern about not being good enough; about poor performance; cannot meet own or other’s expectations | But maybe you put yourself under pressure because you want to do it really well | Clear assignment; Multiple responses allowed |
| D3: Extrinsic Motivation  External pressure, no/ hardly any autonomy, one does the task because it is expected from one, because one has to do it | Tasks don’t make sense, a total scheme aimed at creating work | Clear assignment; Multiple responses allowed |
| D4: Hedonistic Reasons  Not capable of delaying gratification | But you start doing fun things first. | Clear assignment; Multiple responses allowed |
| D5: Poor Competence beliefs  Low self -esteem, no confidence, low self-efficacy | It’s the fear of not being competent enough | Clear assignment; Multiple responses allowed |
| D6: Working conditions  Too many tasks, no individual working places at school | When I want to work on my work schedule I need my PC, in turn this means I need to work from home; Because you always have so many things to do | Clear assignment; Multiple responses allowed |

**How do teachers generally feel when procrastinating?**

| **Category Definition** | **Anchor Examples** | **Coding Rules** |
| --- | --- | --- |
| E1: Positive  General feeling is good/ positive | Initially I would say happy | Clear assignment; Responses mutually exclusive |
| E2: Negative  General feeling is bad/ negative | It’s like your stomach is scrunching up;  Your unhappy with yourself;  It’s not nice having that at the back of your mind | Clear assignment; Responses mutually exclusive |
| E3: Neutral |  | Neither positive nor negative; Clear assignment; Responses mutually exclusive |

**What discrete emotions do teachers feel in the moment of procrastination behavior?**

| **Category Definition** | **Anchor Examples** | **Coding Rules** |
| --- | --- | --- |
| F1: Anxiety | There’s a little anxiety there | Clear assignment; Multiple responses allowed |
| F2: Guilt | I feel guilty | Clear assignment; Multiple responses allowed |
| F3: Depression | I really feel depressed | Clear assignment; Multiple responses allowed |
| F4: Anger | Annoyed at oneself;  Your unhappy and angry on an evening;  I’m angry at myself and at the bulk, because we have large classes | Clear assignment; Multiple responses allowed |
| F5: Joy |  | Clear assignment; Multiple responses allowed |
| F6: Happiness |  | Clear assignment; Multiple responses allowed |
| F7: Unhappiness |  | Clear assignment; Multiple responses allowed |
| F8: Contentment |  | Clear assignment; Multiple responses allowed |
| F10: Shame | And on the other hand I feel a little ashamed | Clear assignment; Multiple responses allowed |
| F9: Disappointment (emerged from interviews) | On the one hand it’s disappointing;  Disappointment because you didn’t do it | Clear assignment; Multiple responses allowed |
| F11: Frustration (emerged from interviews) | Yes, I would call it frustration | Clear assignment; Multiple responses allowed |
| F12: Undefined negative emotion (emerged from interviews) | It’s constricting, it’s like your stomach is scrunching up;  Bad; You always have something at the back of your mind | When participant cannot state clear negative emotions but reports feeling negatively; Multiple responses allowed |
| F13: Defiance (emerged from interviews) | And on the other hand you feel a little defiant | Clear assignment; Multiple responses allowed |

**What consequences do teachers think their procrastination behavior has for them personally and professionally?**

| **Category Definition** | **Anchor Examples** | **Coding Rules** |
| --- | --- | --- |
| G1: Positive Consequence | A positive one;  I’m learning to accept it | General meaning, tends to be overall positive; personally or professionally; Responses mutually exclusive |
| G3: Negative Consequence | I lose the feeling of security;  I don’t feel well;  I have to neglect other things because I initially put it off | General meaning, tends to be overall negative; personally or professionally; Responses mutually exclusive |
| G4: No Consequence | There are no consequences  I didn’t learn from it | Clear statement, no personal AND no professional consequences (positive or negative); Responses mutually exclusive |
