# Supplementary material for: Teacher Procrastination, Emotions, and Stress: A Qualitative Study
Source: Front Psychol. 2019 Oct 11;10:2325. doi: 10.3389/fpsyg.2019.02325 (PMC6798067; doi:10.3389/fpsyg.2019.02325)
Supplement: Supplementary file 2 [file Data_Sheet_2.docx]

Supplementary Material

**Full Interview Guide on Teacher Procrastination**

**Before actual interview commences**

- Introduce yourself as the interviewer
- State the aim of the present study
- Obtain participant’s consent to record the interview
- Inform participant on interview procedure and participant’s rights (e.g. right to withhold answering to specific questions)

**During the interview (start recording)**

- Questions on demographic sample information, including age, gender and years of teaching
- In this interview, we will be talking about procrastination behavior in teachers. Have you heard this term before? As there are many different definitions of procrastination, I will define the one we will be referring to during the interview: Procrastination is the voluntary, needless delay of an intended action despite knowing or expecting to be worse off for the delay, which occurs in a professional academic setting.
- Is this behavior familiar to you?
- When thinking of the different work-related tasks teachers need to do, can you think of tasks you always intend to start but then delay doing them, even though you know you will be worse off for the delay? What tasks would that include? (*If necessary distinguish from other constructs, such as prioritizing*)
- Why do you think you delay working on these tasks? Can you give me any reasons for this?
- How do you generally feel when delaying things you intended to do?
- Think back to a moment when you procrastinated. Can you describe this situation? Can you describe the emotions you experienced in that specific moment when you intended to work on a certain task and then decided to delay working on it?
- Does this behavior have any consequences for you, either personally or professionally? If it does, are these consequences positive or negative?
- How stressful is procrastinating on work-related tasks on a scale of 1 to 7 for you? (1 being not stressful and 7 being extremely stressful)

**At the end of the interview**

- Thank participants for their time and contribution
- Ask if participant would like to add anything
- Ask if participant has any questions

**Stop recording**

**General Note:** Examples may be given, if participant has difficulty understanding or answering the questions
